# Equivalent VTE rates after total joint arthroplasty using thromboprophylaxis with aspirin *versus* potent anticoagulants: retrospective analysis of 4562 cases across a diverse healthcare system

**DOI:** 10.1186/s42836-021-00101-8

**Published:** 2021-12-03

**Authors:** Chelsea Matzko, Zachary P. Berliner, Gregg Husk, Bushra Mina, Barton Nisonson, Matthew S. Hepinstall

**Affiliations:** 1 Department of Orthopedic Surgery, Lenox Hill Hospital, Northwell Health, New York, New York USA; 2 Department of Orthopedic Surgery, Boston University Medical Center, Boston, Massachusetts USA; 3 Department of Orthopedic Surgery, NYU Langone Health, 301 E 17th St, Suite 1402, New York 10003 New York, USA

**Keywords:** Aspirin, Venous thromboembolism, Deep venous thrombosis, Prophylaxis, Thromboprophylaxis, Anticoagulant, Anticoagulation, Hip arthroplasty, Knee arthroplasty

## Abstract

**Background:**

Guidelines support aspirin thromboprophylaxis for primary total hip and knee arthroplasty (THA and TKA) but supporting evidence has come from high volume centers and the practice remains controversial.

**Methods:**

We studied 4562 Medicare patients who underwent elective primary THA (1736, 38.1%) or TKA (2826, 61.9%) at 9 diverse hospitals. Thirty-day claims data were combined with data from the health system’s electronic medical records to compare rates of venous thromboembolism (VTE) between patients who received prophylaxis with: (1) aspirin alone (47.3%), (2) a single, potent anticoagulant (29%), (3) antiplatelet agents other than aspirin or multiple anticoagulants (21.5%), or (4) low-dose subcutaneous unfractionated heparin or no anticoagulation (2.2%). Sub-analyses separately evaluating THA, TKA and cases from lower volume hospitals (*n* = 975) were performed.

**Results:**

The 30-day VTE incidence was 0.6% (29/4562). VTE rates were equal in patients receiving aspirin and those receiving a single potent anticoagulant (0.5% in both groups). Patients with VTE were significantly older than patients without VTE (mean 76.5 *vs*. 73.1 years, *P* = 0.04). VTE rate did not associate with sex or hospital case volume. On bivariate analysis considering age, aspirin did not associate with greater VTE risk compared to a single potent anticoagulant (OR = 2.1, CI = 0.7–6.3) with the numbers available. Odds of VTE were increased with use of subcutaneous heparin or no anticoagulant (OR = 6.4, CI = 1.2–35.6) and with multiple anticoagulants (OR = 3.6, CI = 1.1–11.2). THA and TKA demonstrated similar rates of VTE (0.5% *vs*. 0.7%, respectively, *P* = 0.43). Of 975 cases done at lower volume hospitals, 387 received aspirin, none of whom developed VTE.

**Conclusions:**

This study provides further support for aspirin as an effective form of pharmacological VTE prophylaxis after total joint arthroplasty in the setting of a multi-modal regimen using 30-day outcomes. VTE occurred in 0.7% of primary joint arthroplasties. Aspirin prophylaxis did not associate with greater VTE risk compared to potent anticoagulants in the total population or at lower volume hospitals.

## Background

The risk of postoperative venous thromboembolism (VTE), including deep vein thrombosis (DVT) and pulmonary embolism (PE) following “major” orthopaedic surgery (total joint arthroplasty or hip fracture repair) is well established [1]. Following recognition of this risk, the use of potent anticoagulants for chemoprophylaxis became common after total joint arthroplasty (TJA) and has long been endorsed by the American College of Chest Physicians (ACCP) guidelines [2]. Routine chemoprophylaxis, mechanical prophylaxis, regional anesthesia, decreased narcotic use and early postoperative ambulation protocols have contributed to a decline in VTE rates with modern TJA [3, 4].

Specific high-volume arthroplasty centers have used multi-modal VTE prophylaxis protocols incorporating aspirin rather than potent anticoagulants for many years and reported low rates of VTE [1, 3–7]. Nevertheless, despite decades of use of aspirin as a cost-effective and safe VTE prevention tool, with efficacy demonstrated even in patients considered to be at high risk for VTE [8], use of aspirin for VTE prophylaxis in orthopedic patients has remained controversial.

The rate of pulmonary embolism after TJA has unfortunately not decreased significantly over past decades [9]. Indeed, it is notable that potent anticoagulants have been associated with lower incidence of surrogate endpoints such as asymptomatic DVT, but not a reduction in the most patient-important endpoints, symptomatic and fatal PE [6, 10, 11]. The persistence of fatal PE has been interpreted by some to support the need for potent anticoagulants [12], whereas others have argued that the persistent incidence of PE proves potent anticoagulants are no more effective than aspirin in preventing patient-important complications [13, 14]. Compared to aspirin, potent anticoagulants have been associated with increased bleeding risk, as well as postoperative wound complications and increased length of stay [15–19]. However, there is a great deal of variability in the definition of major bleeding in VTE prevention trials for hip and knee arthroplasty. Some trials excluded surgical site bleeding, resulting in highly variable rates of major bleeding between studies with similar treatment regimens and patients [20].

In 2011, American Academy of Orthopaedic Surgeons (AAOS) endorsed aspirin as one of several evidence-based forms of thromboprophylaxis for total joint arthroplasty (TJA) [11] and in 2012, ACCP guidelines similarly endorsed aspirin as safe and effective VTE prophylaxis following total joint arthroplasty (TJA) in properly selected patients [21]. Subsequently, the use of aspirin for VTE prophylaxis following TJA has increased markedly [22]. Nevertheless, there remain concerns that aspirin may be less effective than potent anticoagulants, resulting in the funding of an ongoing large prospective, multi-center randomized clinical trial (RCT) attempting to provide higher-level evidence to resolve this controversy, but these results are not yet available.

Much of the data informing the recommendations endorsing the safety and efficacy of aspirin thromboprophylaxis came from high-volume centers [7, 21, 23–28]. Furthermore, subsequent studies validating the efficacy of aspirin following TJA have also primarily been performed at high-volume centers [5–7, 29]. Given the suspected relationship between surgical efficiencies and VTE risk, the question as to whether aspirin is equally safe and effective for VTE prophylaxis after TJA in diverse community settings remains unresolved.

We sought to evaluate whether the safety and efficacy of aspirin VTE prophylaxis documented at high volume centers could be extrapolated to Medicare patients across a spectrum of practice environments. We hypothesized that VTE risk would not differ between aspirin and potent anticoagulants across facilities. Therefore, we retrospectively analyzed data from across a large and diverse health care system to identify any interaction between hospital TJA volume, chemoprophylaxis choice and VTE rates.

## Materials and methods

We retrospectively reviewed the data from 4562 Medicare patients who underwent a total hip arthroplasty (1736, 38%) or total knee arthroplasty (2826, 61.9%) at 9 diverse hospitals across one North American health system from January 2014 to October 2017 (Table 1). We excluded patients receiving arthroplasty for hip fracture, owing to the differential rates of comorbidities and complications in this population [30–32]. VTE prophylaxis was determined based on surgeon preference and medical judgment at each hospital. The treatment duration was not standardized. Many surgeons routinely prescribed 4 weeks of therapy and most surgeons prescribed 2 to 6 weeks of therapy. Data were collected from the patients of all 141 orthopedic surgeons who performed total hip or knee arthroplasty during the designated time period. Hospitals within the system were both urban and suburban, ranging from 239 beds to 806 beds, with and without teaching programs. Concomitant mechanical prophylaxis with compression devices was standard but was not systematically recorded. All hospitals participated in CJR or BPCI, enabling access to data from the Center for Medicare & Medicaid Services (CMS) that included care outside our health system. Hospitals were stratified by elective arthroplasty volume into either the “higher volume” or “lower volume” category for analysis. Hospitals with fewer than 400 primary hip or knee arthroplasty cases in Medicare patients during the 3.8-year study period were considered lower volume. There were three higher-volume hospitals and six lower-volume hospitals. Sample size was 3587 cases in the higher-volume hospital cohort (79% of the cases studied) and 975 cases in the lower-volume hospital cohort (21% of the cases studied).

**Table 1:** Comparison of demographics and length of stay between anticoagulant groups

	Aspirin Alone	Single Potent AC	Antiplatelet/ Multiple AC	SQ heparin/ No AC	Total cohort
Age (years), mean (SD)	73.6 (7.4)	72.7 (7.8)	75.2 (7.4)	72.8 (9.6)	73.6 (7.6)
Female, %* (*n*)	47% (1448)	71% (957)	58% (563)	70% (70)	67% (3048)
Length of stay, mean (SD)	4.0 (1.2)	4.4 (1.6)	4.4 (1.4)	4.6 (2.6)	4.2 (1.4)
Total hip arthroplasty, %** (*n*)	44.1% (951)	30.7% (406)	34.8% (341)	37.6% (38)	38.1% (1736)
Total knee arthroplasty, %** (*n*)	55.9% (1207)	69.3% (916)	65.2% (640)	62.4% (63)	61.9% (2826)

The anticoagulant and/or antiplatelet medication(s) administered postoperative day one were collected from the system-wide electronic medical record. Four prophylaxis categories were defined: (1) aspirin alone, (2) a single potent anticoagulant (*i*.*e*. low-molecular-weight heparin, warfarin, factor X_a_ inhibitor), (3) an anti-platelet agent other than aspirin *or* multiple anticoagulants, and (4) low-dose subcutaneous unfractionated heparin alone *or* no anticoagulation. Patients were evaluated for VTE at the discretion of their treating clinicians. 30-day Medicare claims data were reviewed to identify any new postoperative VTE diagnoses that occurred during this period, which was recognized to be the period of highest risk for VTE after TJA. Claims data included diagnoses made in the outpatient setting and those made outside our health system. Where possible, VTE findings obtained from CMS claims data were confirmed using review of the health system Electronic Health Records. This allowed elimination of mis-coded events, such as those that occurred preoperatively and presumptive clinical diagnoses that were refuted by diagnostic testing. The study was approved by the health system’s Institutional Review Board.

### Statistical analysis

Statistical analyses were performed with IBM SPSS version 25 (Armonk, NY, USA). Independent variables included anticoagulant agents, age, gender, procedures (total hip arthroplasty* vs*. total knee arthroplasty), high- *vs*. low-volume center, and length of stay. High-volume centers were defined as those that performed more than 400 primary arthroplasty procedures during the 3.8-year study period. The primary outcome was occurrence of VTE.

An independent sample’s *t*-test was performed for continuous variables. Chi-square or Fisher’s exact test was performed for categorical variables. A multi-variable Logistic regression was performed to evaluate the relationship between anticoagulant, demographics, and the primary outcome of VTE. Demographic variables that associated with incidence of VTE on univariable testing were entered into the multi-variable model.

A secondary analysis focusing on the patient population treated at lower-volume hospitals was performed to investigate the generalizability of findings at higher-volume hospitals to this practice setting. For all analyses, significance level was set at *P* <  0.05.

## Results

Overall, VTE incidence was low, standing at 0.6% (29 of 4562) in the 30-day post-operative period following total joint arthroplasty. Patients with VTE were significantly older than their non-VTE counterparts (mean age 76.5 *vs*. 73.1 years, *P* = 0.04) (Table 2). There was a 0.5% (9/1736) incidence of VTE in the total hip arthroplasty group, and a 0.7% (20/2826) incidence of VTE in the total knee arthroplasty group (*P* = 0.43).

**Table 2:** Demographics and length of stay associated with incidence of VTE

	No VTE (***n*** = 4505)	VTE (***n*** = 29)	***P*** value
Age (years), mean (SD)	73.1 (7.6)	76.5 (9.1)	0.04
Female, % (n)	67% (3027)	72.4% (21)	0.52
Length of stay, mean (SD)	4.2 (1.4)	6.9 (4.3)	< 0.001

On univariate analysis (Table 3), patients receiving aspirin prophylaxis had a VTE rate equivalent to that of the cohort receiving a single potent anticoagulant (0.5% in both cohorts) (Table 3). VTE rate was significantly higher in the 101 patients receiving subcutaneous heparin or no anticoagulant (2.0%, *P* <  0.05). VTE rate was also increased in the 981 patients receiving either anti-platelet agents other than aspirin or multiple anticoagulants (1%, *P* < 0.05).

**Table 3:** Venous thromboembolism rates for each prophylaxis category and surgical procedure

	Percent of population	VTE Rate
Aspirin alone, % (*n*)	47.3% (2158)	0.5% (11)
Single potent anticoagulant (low-molecular-weight heparin, warfarin, factor X_a_ inhibitor), % (*n*)	29.0% (1322)	0.5% (7)
Anti-platelet agents other than aspirin/ multiple anticoagulants, % (*n*)	21.5% (981)	1.0% (9)
Low dose subcutaneous unfractionated heparin alone or no anticoagulation, % (*n*)	2.2% (101)	2.0% (2)
Total hip arthroplasty, % (*n*)	38% (1736)	0.5% (9)
Total knee arthroplasty, % (*n*)	62% (2826)	0.7% (20)

Neither joint replaced (hip *vs*. knee) nor patient gender associated with VTE on univariate testing. Although length of stay associated with VTE rate, this is not an independent variable; in-hospital VTE can increase length of stay. Therefore, only age and anticoagulant category were included in multivariable testing. When controlling for age, use of aspirin did not associate with significantly greater VTE risk compared to a single potent anticoagulant (OR = 2.1, 95% Confidence Interval (CI) = 0.7–6.3), but odds of VTE were increased with use of subcutaneous heparin or no anticoagulant (OR = 6.4, CI = 1.2–35.6) and with multiple anticoagulants (OR = 3.6, CI = 1.1–11.2). Age persisted as a risk factor in this model (OR = 1.06, CI = 1.0–1.1).

Though hospital case volume differed across the health system, VTE incidence did not significantly differ between high- and low-volume centers. The incidence of symptomatic VTE was 0.5% (5/975 cases) across the 6 lower-volume hospitals, which did not differ significantly from the 0.7% rate of symptomatic VTE across the 3 higher-volume hospitals (24/3587 cases; *P* = 0.59). Of the 975 cases done at lower-volume hospitals, none of the 150 patients who were prescribed aspirin developed VTE, while 1 VTE occurred in 462 patients who received potent anticoagulants.

## Discussion

Across a large and diverse healthcare system, there was a low 0.6% incidence of VTE in the first 30 days after primary TJA in Medicare patients. Aspirin prophylaxis was associated with a low VTE risk similar to use of a single potent anticoagulant following total hip and knee arthroplasty. Based on data from our system-wide EMR, combined with claims data from Medicare to help capture events outside our health system, this observation was not confined to high-volume centers and indeed held true in the subgroup of patients treated at hospitals with lower volumes of elective hip and knee arthroplasty. Widespread use of aspirin chemoprophylaxis in nearly 50% of patients was associated with a low VTE event rate of 0.5%, which was equivalent to the 0.5% rate observed in patients receiving a potent anticoagulant. VTE rates were not significantly different in patients undergoing THA *vs*. TKA (0.5% *vs*. 0.7%, respectively). This study provides additional evidence that aspirin is safe and effective for use in “standard risk” patients following primary TJA.

Our study has several limitations. Our methodology did not allow us to reliably determine the intended treatment duration, or any medication changes that may have occurred in the outpatient setting. We analyzed 30-day rather than 90-day outcomes. While VTE may occur up to 90 days after TJA, the majority occur within the initial 30 days [33]. Although we found identical rates of VTE with use of aspirin *vs*. potent anticoagulant on univariate analysis, and found no statistically significant difference in odds of VTE on multivariate analysis, – the 95% confidence interval for the odds ratio (0.7–6.7) included both a small difference favoring aspirin and a larger difference favoring potent anticoagulants. Thus we cannot exclude the possibility that potent anticoagulants might be more effective, particularly in patients at higher risk of VTE. Nevertheless, the fact that our study of 4562 hip and knee arthroplasties could not detect a difference in efficacy between the two most commonly used thromboprophylaxis strategies reflects an extremely low incidence of VTE in modern arthroplasty practice, allowing consideration of differences in risk, cost and patient acceptance.

Our study is by no means the first to compare VTE rates with use of aspirin *vs*. potent anticoagulants after total joint arthroplasty. Our findings should therefore be considered in the context of other published data. A recent meta-analysis [34] of 13 randomized controlled trials with 20,115 patients found aspirin to be associated with a significant reduction in VTE compared to placebo, with a non-significantly lower rate of VTE compared to other methods of thromboprophylaxis. Only 4 of the included studies examined a potent anticoagulant such as warfarin or low molecular weight heparin, however. Furthermore, the rigidly defined exclusion criteria associated with randomized controlled trials may have excluded the patients most likely to experience a thromboembolic event. This concern supports the need for pragmatic studies investigating aspirin thromboprophylaxis as used outside clinical trials. While much of the existing pragmatic evidence supporting aspirin thromboprophylaxis has come from specific high-volume arthroplasty centers [7, 21, 23–28], there are limited data available from more diverse settings. Our retrospective data therefore add to the literature, demonstrating aspirin prophylaxis to be safe and effective with VTE rates comparable to use of a single potent anticoagulant when used in just over 50% of elective hip and knee arthroplasty cases across a large and diverse health system.

Patients receiving only subcutaneous heparin or no anticoagulant in the first 24 h following surgery showed higher VTE risk than those receiving aspirin or a single potent anticoagulant. This observation is consistent with the findings of the recent meta-analysis referenced above [34]. Patients in whom both aspirin and potent anticoagulants are contraindicated should be educated about this increase in VTE risk and non-pharmacologic strategies should be employed to reduce risk whenever possible. Older patients also demonstrated a significantly higher risk for VTE, a finding which coincides with national trends seen in DVT rates [35]. Our detection of these established differences may be viewed as supporting the sensitivity of our methodology to identify large, clinically-important differences in risk associated with anticoagulant strategies and patient demographics.

Patients receiving non-aspirin antiplatelet agents or multiple anticoagulants showed higher VTE risk than those receiving aspirin alone. It seems unlikely that multiple anticoagulants increase VTE risk, so this finding may reflect selection bias, with higher preoperative risk in those receiving multiple anticoagulants. As such, this finding highlights a limitation of our risk stratification. Although we included patient age in a multivariate analysis, our analysis was unable to control for specific comorbidities including personal history of VTE. Given the retrospective nature of this study and the limited risk stratification, our results do not establish that aspirin is equally effective to potent anticoagulants for VTE prophylaxis across all patient populations, nor does it address the question as to whether potent anticoagulants might offer an advantage in a select high risk population.

It should be noted that our data do not allow us to specifically evaluate the safety and efficacy of ASA in high-risk patients. Huang *et al* demonstrated aspirin to be as effective as warfarin for VTE prophylaxis in higher-risk patients, defined as those with history of VTE, active malignancy, COPD, pulmonary hypertension, stroke or a combination of lesser risk factors including advanced age, anemia, congestive heart failure, peripheral vascular disease or history of myocardial infarction [8]. Others have advocated selective use of potent anticoagulants in patients with preexisting conditions such cancer, sepsis, hypercoagulability, with multiple studies showing that prospective risk stratification and individualized risk modeling can predict VTE and symptomatic PE risk [19, 36]. Nevertheless, our data support the emerging consensus that aspirin offers safe and effective VTE chemoprophylaxis in patients determined to be standard or low risk [8, 37].

Ultimately, determining the best VTE prophylaxis regimen for a given patient is difficult and multiple factors deserve consideration including thrombotic risk, bleeding risk, medication interactions and comorbid medical conditions. Our study did not compare bleeding or other adverse events between anticoagulants, but prior data have shown a lower incidence of periprosthetic joint infection and mortality with aspirin prophylaxis following total joint arthroplasty, when compared to patients who received warfarin [8].

Our study focused on the Medicare population, utilizing data made available through participation in CMS bundled payments programs. As such, caution should be exercised when generalizing these findings to younger, commercially-insured patients. The large number of surgeons included working across 9 different centers nevertheless increases the generalizability of our findings to diverse practice settings and suggests that our results are not specific to one surgical technique, patient population or specific prophylaxis protocol.

Retrospective review of outcomes associated with non-randomized treatment allocation is always vulnerable to selection bias and we look forward to the results of ongoing multicenter prospective randomized controlled clinical trials. For example, the PEPPER trial is an ongoing randomized, prospective study comparing the effectiveness of Pulmonary Embolism Prevention After Hip and Knee Replacement with aspirin, warfarin and rivaroxaban. Nevertheless, as the modern event rate for VTE is thankfully low, well-powered prospective studies are costly and take years to complete. For the time being, retrospective studies provide necessary data to inform clinical decision making.

## Conclusions

This study provides further support for aspirin as an effective form of pharmacological VTE prophylaxis after total joint arthroplasty in the setting of a multi-modal regimen including preferential use of regional anesthesia, rapid mobilization and sequential compression devices during the hospital stay. In this setting, the rate of venous thromboembolism in patients receiving aspirin was equal to that in patients receiving more potent anticoagulants following total joint arthroplasty.

## Data Availability

The datasets used and/or analyzed during the current study are available from the corresponding author on request.

## Abbreviations

THA: Total Hip Arthroplasty
TKA: Total Knee Arthroplasty
VTE: Venous Thromboembolism
DVT: Deep Vein Thrombosis
PE: Pulmonary Embolism
TJA: Total Joint Arthroplasty
ACCP: American College of Chest Physicians
